# Neurosensory Affectation in Patients Affected by Wolfram Syndrome: Descriptive and Longitudinal Analysis

**DOI:** 10.3390/healthcare11131888

**Published:** 2023-06-29

**Authors:** Gema Esteban-Bueno, Aída María Berenguel Hernández, Nicolás Fernández Fernández, Miguel Navarro Cabrero, Juan R. Coca

**Affiliations:** 1 Clinical Management Unit Almería Periphery—Almería Health District, Andalusian Health Service, 04120 Costacabana, Almería, Spain; 2 Spanish Association for the Research and Support of Wolfram Syndrome, 04120 Costacabana, Almería, Spain; 3 Clinical Genetics Biogenox, 04720 Aguadulce, Almería, Spain; aidaberenguel@biogenox.es; 4 La Inmaculada Hospital, Andalusian Health Service, 04600 Huércal-Overa, Almeria, Spain; 5 Social Research Unit on Health and Rare Diseases, Department of Sociology and Social Work, University of Valladolid, 42004 Soria, Castile and León, Spain; juanr.coca@uva.es

**Keywords:** Wolfram syndrome, DIDMOAD, hearing loss, hearing impairment, genetic mutation

## Abstract

Wolfram syndrome (WS) is a rare neurodegenerative disease usually of autosomal recessive origin. There is limited research about sensorineural hearing loss, despite it being a fundamental entity. It is important to broaden the study of this disease and specify a set of tests required for an adequate assessment of patients for efficient monitoring and control. The fundamental objective of this research is to understand WS from a biomedical perspective in order to help in its diagnosis, follow-up, and control. Pure tones audiometry, tympanometry, speech perception, the speech intelligibility index without aid, and testing at high frequencies were among the audiological measurements utilised since they were deemed suitable for standardised follow-up. Mixed linear models were used to examine the effects of age, time, or mean interaction in pure-tone (IPT), the average of high frequencies (HFA), auditory brainstem response (ABR), and brainstem auditory evoked potentials (BAEP). The genetic analysis allowed mutations to be classified into three phenotype-genotype groups, where the phenotype indicated the severity of the hearing loss. Patients with homozygous gene changes had a more severe neurosensory phenotype. The early discovery of sensorineural hearing loss and WS is crucial since it allows intensive follow-up and treatment of the person affected from the start.

## 1. Introduction

Wolfram syndrome (WS) is a genetically based neurodegenerative disease. It is characterized by the onset of diabetes mellitus (DM) associated with optic atrophy (OA). This association between both manifestations occurs at an early age (before 17 years of age). In addition, the people affected by this illness often present with diabetes insipidus (DI), hearing loss, and neurodegeneration. It was first described in 1938 by Wolfram and Wagener, who found four of eight siblings with juvenile diabetes mellitus and optic nerve atrophy [1,2]. WS presents other less common manifestations, such as renal tract abnormalities, neuropsychiatric disorders, and hypogonadism [1]. This syndrome is also known by the acronym DIDMOAD, which corresponds to the initials in English of the characteristic elements of the classic phenotype of the disease: DI, DM, OA, and sensorineural deafness (D) [3,4,5]. It is a very rare disease with an estimated prevalence of 1 in 770,000 in the UK and 1 in 100,000 in North America, and a carrier frequency of 1 in 354 [3,4].

WS presents a variable clinical picture, depending on the penetrance of the altered gene. It is usually caused by mutations in the WFS1 gene on chromosome 4p16.1. There is a less frequent variant due to mutations in the CISD2 gene [1,6] called WS type 2. Therefore, depending on the causative gene, the existence of two types of WS has been established: type I and type II. Type I is identified by the classic form of the complaint and is caused by autosomal recessive mutations of the WFS1 gene. However, type II is linked to mutations in CIDS2 and has the characteristics of WS1 but does not present with diabetes insipidus; it is characterized by the appearance of bleeding peptic ulcers and irregular platelet aggregation at an early age (6 years old) [6]. Although this is still under debate, some authors claim that WS is a pathology that follows an autosomal recessive inheritance pattern [5,6], but there are reported cases of WS1 with an autosomal dominant character due to certain missense mutations in exon 8, which are responsible for the so-called Wolfram-like syndrome (WLS) [5].

**Figure 1:**
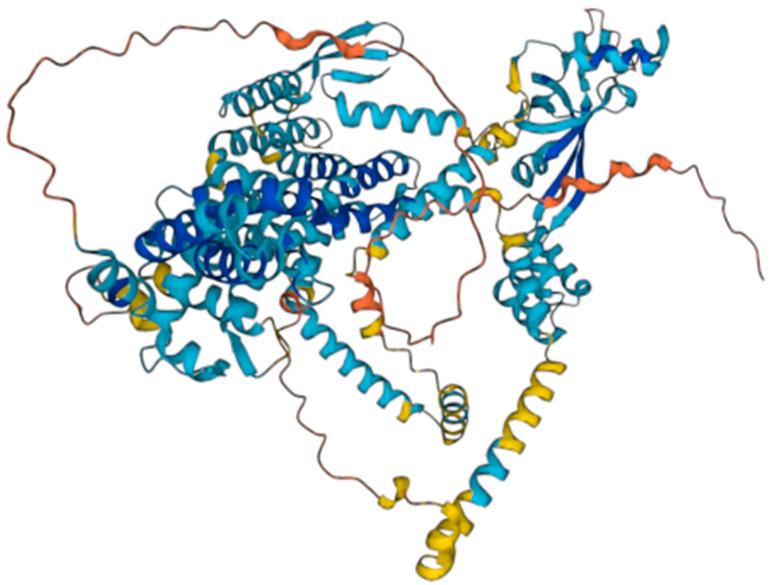
Wolframin structure. Source: AlphaFold Protein Structure Database.

The WFS1 gene encodes for the wolframin protein. Wolframin has three structural domains: an amino-terminal hydrophilic region of approximately 300 residues, a carboxy- terminal hydrophilic region of approximately 240 residues, and a central hydrophobic region of approximately 350 residues containing 9–10 transmembrane segments [7]. According to the AlphaFold database [8,9], wolframin has 890 amino acids and is predicted to have a transmembrane structure composed of nine alpha helices (transmembrane segments) [10] (Figure 1). Wolframin is, therefore, a transmembrane protein that is present in various tissues of the human body. It participates in the regulation of cellular Ca^2+^ homeostasis since it is involved in the modulation of the filing state of the Ca^2+^ deposit of the endoplasmic reticulum. In this sense, it negatively regulates the stress response of the endoplasmic reticulum and it increases the stability of the ATP6V1A and ATP1B1 subunits of V-ATPase [11]. Currently, the function of wolframin in the ear has not yet been established, but it has been described as being involved in the ionic exchange at the level of the inner ear [8]. In addition, we know that alterations in this protein pose a high risk of presenting sensorineural hearing loss [12].

Clinically, WSL is defined by low-frequency sensorineural hearing loss (LFSNHL) (also known as DFNA6/14/38 LFSNHL), optic nerve atrophy, the onset of psychiatric disorders (anxiety, depression, or hallucinations), and the absence of DM [6,13]. It can also cause isolated non-syndromic low-frequency sensorineural hearing loss and, occasionally, isolated DM [14]. Nowadays, mortality occurs before the fifth decade of life [4,15], the main cause being central nervous system disorders.

In general, there are limitations in the studies of rare diseases due to the scarcity of cases, the discontinuity in follow-ups, and their high mortality. Specifically, in the case of WS, there are few descriptive studies with a sufficient population that use standardized audiometric measurements [16,17]. In the same way, the studies with a population volume that assess the patient from a multidisciplinary perspective are minimal, preventing the establishment of conclusions about a possible correlation between genetics and other aspects, such as the sex of the patients or the cardinal symptoms of WS. For this reason, regarding the diagnosis of WS1, there is not a main marker, but there are clinical diagnostic criteria, such as progressive OA before 17 years of age and juvenile DM developed before 15 years of age [12].

Due to its clinical significance, this study extends the knowledge of the affectation and evolution of the auditory nerve. This is significant, as hearing is regarded as one of the primary components of this syndrome.

The objective is, therefore, to improve the diagnosis and knowledge of WS1, focusing on the audiological assessment of patients, their genetic characteristics, and their evolution.

## 2. Materials and Methods

This research has analyzed the neurosensory involvement of Spanish and Portuguese patients affected by Wolfram syndrome. The empirical analysis was mixed, employing a documentary methodology (review of medical records) and a series of quantitative (audiometry, impedance audiometry) and qualitative (intelligibility test) analyses of hearing measurement. The methodological strategy consisted of a descriptive and longitudinal analysis of WS focused on auditory impairment.

### 2.1. Participants

The individuals participating in this study were recruited sequentially from 1999 to 2021. The initial population sample (referred to herein as population 1) had a total of 68 patients. The study population is limited to the Spain–Portugal area. There are three cases of people from Latin America. The inclusion criteria for population 1 were as follows: having the diagnosis of Wolfram syndrome, patient accessibility, and mutations in the gene coding WS. The 68 patients were given a general evaluation for the affectation of hearing. Population 1 included 42 men and 26 women, with a mean age at the start of follow-up of 19.79 years, a standard deviation (SD) of 10.25 years, and a range in age between 4 years and 60 years.

Likewise, a subgroup of 36 patients (population 2) was selected to undergo a more in-depth audiological assessment between 2011 and 2021. For this study, an annual assessment was carried out in which the patients were attended in person by our multidisciplinary team. Due to events unrelated to the research group, not all the patients were able to attend all of the face-to-face visits. The sample of the 36 patients attended personally and periodically includes 14 men (38.88%) and 22 women (61.11%), with ages ranging from 8 to 56 years at the beginning of the follow-up. The inclusion criteria for population 2 were the following: having the diagnosis of Wolfram syndrome with genetic confirmation that it affected the WFS gene, being able to perform audiological tests for several years to determine the evolution of the disease, and agreeing to be part of the study.

The connection with the patients was carried out through an active search for cases, contacting different health centers where there could be cases of WS, or reaching out to social entities that care for people with deaf–blindness difficulties. The public consolidation of the research team made it easier for the patients’ referring physicians to communicate the cases to us and resulted in several patients who required follow-up when they contacted the study group through social media. All of this reveals that the patients were included by means of ad hoc pre-selection. Patients who were not affected by Wolfram syndrome, did not provide informed consent, had insufficient data, or who did not wish to participate in the study were excluded (exclusion criteria).

### 2.2. Ethical Considerations

The privacy and confidentiality of all the subjects were preserved in accordance with Organic Law 03/2018, of 5 December, on data protection (OLDP). Alphanumeric codes were used to identify the patients to ensure anonymity. All the patients gave their verbal informed consent for participation in the study. In the case of children under 18 years of age or legally incapacitated persons, their legal guardians were the ones who gave their consent for their participation. Likewise, the study was conducted in accordance with the Declaration of Helsinki and was approved by the Ethics Committee of Research Almería Center (protocol code wolfram15 on 29 April 2016).

### 2.3. Procedures

From population 1, the sociodemographic data and the age of onset of all WS symptoms and their evolution were collected from the patients’ clinical history, with special interest in the 4 cardinal symptoms (DM, DI, AO, D) and in the involvement of the WS. At the same time, the genetic information contained in their history was obtained. This data allowed us to carry out a follow-up of the cases and a statistical analysis, as well as a descriptive analysis of the mutations and the frequency of appearance of the different genetic alterations.

From population 2 (who attended in person), the information described above was collected for a total of 68 patients. In addition, a series of specific assessments were carried out [12] to complement the previous information. During the initial examination, an otoscopy was performed since it allows for an assessment of the middle ear and the existence of earwax plugs, which, if visualized, can be removed for better assessment. Subsequently, the following evaluations were made:

1. Pure-tone audiometry: This test is used to detect hearing loss in these patients, which is key for any type of hearing loss. Frequencies ranging from 125 Hz to 8000 Hz are measured, providing the hearing status of the patients.
2. Impedance tympanometry: This test analyzes the tympanic compliance.
3. High-frequency audiometry: This test is similar to pure tone audiometry, although it records higher frequencies. Similar to other diseases in the inner ear, high frequencies are altered sooner, allowing an earlier detection of hearing loss as compared to traditional audiometry. As a result, it can be employed in the early stages of diagnosis; however, this audiometry is not available in all centers.
4. Intelligibility tests: These tests, such as speech audiometry, permit doctors to determine whether the patient, in addition to hearing, is able to understand words. They are useful, among other things, in determining if the patient can benefit from a hearing aid.
5. Auditory brainstem response (ABR), also known as brainstem auditory evoked potentials (BAEP): Evoked potentials are an objective test that indicates the integrity of the auditory pathway. They are very useful in children because they do not require the collaboration of the patient. They usually give results that are consistent with audiometry, which is why, in many studies, they directly perform audiometry, which is simpler and faster.

### 2.4. Instrumentation

The liminal tonal audiometry, the speech audiometry, and the open field were performed in a room of a cabin made up of wood and cork with an acoustic treatment made to measure for the ENT service of our hospital; this room creates very important acoustic isolation. These tests were performed with an AD629 interacoustic audiometer. The otoscopy was performed with a Riester e-scope^®^ otoscope (Rudolf Riester GmbH, Jungingen, Germany) and if earwax was visible, the ears were cleaned with the help of a Leica M320 microscope plus the relevant material (curettes, scissors, aspirator connected to a vacuum, etc.). Tympanometric measurements were obtained with an Interacoustics AT22t tympanometer and an AT235 tympanometer. The high frequency was achieved with the AD629 model audiometer, and the BAEP were assessed with the interacoustic Eclipse.

### 2.5. Analysis of Data

A descriptive statistical analysis of the variables was carried out using the IBM SPSS version 26.0 statistical package. Data representation was carried out using the Prism version 8.0 program.

## 3. Results

### 3.1. Population 1

The analysis of all the study subjects with WS and genetic confirmation (mutation in the WFS1 gene) showed that 80.89% (*n* = 55) suffered from hearing impairment and only 19.11% (*n* = 13) did not. Neurosensory alteration was present in all cases in which hearing was affected. Of those who suffered from hearing impairment, 100% had neurosensory impairment and only one of them also presented impaired conduction due to earwax in the ear canal. The mean age of onset of hearing impairment was 15 years (SD = 8.09 years). In the global sample of 68 patients, of the 55 (80.88%) who suffered from hearing loss, it was determined that 40% (*n* = 22) were less than or equal to 12 years of age, 25.45% (*n* = 14) were between 13 and 18 years old and, finally, 34.54% (*n* = 19) were 18 or older. Regarding vision, 100% (*n* = 68) suffered from OA. The mean age of onset was 10 years (SD = 5.03 years), 73.53% (*n* = 50) of the patients were 12 or younger, 19.12% (*n* = 13) were between 13 and 18, and 7.35% (*n* = 5) were 18 years of age or older.

After analyzing the clinical histories, it was confirmed that most of the patients had type 1 DM. In fact, of the 68 patients, 98.53% (*n* = 67) presented type 1 DM. The mean age of onset was 5.5 years (SD = 3.33 years). In total, 95.52% (*n* = 64) of the patients were 12 years of age or younger, 4.47% (*n* = 3) were between 13 and 18 years of age, and none presented after 18 years of age. Regarding ID, of the 68 patients, 55.88% (*n* = 38) manifested it. The mean age of onset was 10.99 years (SD = 5.32 years). A total of 65.79% (*n* = 25) of the patients were 12 years old or younger, 21.05% (*n* = 8) were between 13 and 18 years old, and 13.15% (*n* = 5) were 18 years old or older. Of the 68 patients in the sample, only 9 presented two simultaneous entities on examination. The rest presented 1 of the 4 main criteria of the disease. The type 1 DM that the patients presented is characterized, in all cases, by not being of autoimmune origin and being negative for HLA DR3 and DR4, in addition to lacking the antibodies that usually appear in type 1 DM. In addition, it also shows specific clinical characteristics, such as not presenting a tendency to ketosis, the insidious onset in all cases, and the scarce presence of acute and chronic complications. Regarding ID, of the 68 patients, 55.88% (*n* = 38) manifested it. The mean age of onset was 10.99 years (SD = 5.32 years). A total of 65.79% (*n* = 25) of the patients were 12 years of age or younger, 21.05% (*n* = 8) were between 13 and 18, and 13.15% (*n* = 5) were 18 years or older.

Based on the symptoms with which the syndrome begins, the majority of patients (*n* = 47) presented with type 1 DM at a mean age of onset of 4.69 years (SD = 2.78), with the diagnosis of WS being given at 16.81 years (SD = 9.29 years) with a diagnosis delay of 12.12 years (SD = 8.90). Vision was the presenting symptom in 9 patients at a mean age of 5.56 years (SD = 2.19 years), obtaining the diagnosis of WS at 13.78 years (SD = 6.48 years). In this case, the diagnosis delay is 9.38 years (SD = 5.90). The primary symptom of sensorineural deafness from birth was found in 2 patients. In these cases, the diagnosis of WS was made at 8 years and 9 years, respectively, generating a diagnostic delay of 8.5 years (SD = 0.71 years). Only 1 patient presented with isolated DI at a mean age of onset of 4 years, being diagnosed with WS at 27 years of age. Five patients presented two entities at debut, jointly presenting type 1 DM and optic nerve atrophy, at a mean age of onset of 5.15 years (SD = 3.77 years), with the diagnosis of WS at 12.60 years (SD = 4.67 years) with a diagnosis delay of 7.45 years (SD = 7.63 years).

In 3 patients, type 1 DM associated with DI were the presenting entities at a mean age of onset of 5.33 years (SD = 1.15 years), with the diagnosis of WS being given at 25.33 years (SD = 2.31 years) with a diagnosis delay of 20 years (SD = 2.0 years). The association of optic atrophy with deafness as presenting entities occurred in only one 10-year-old patient, with the diagnosis of WS being given at 10 years. In this case, a delay in diagnosis has not been obtained. A total of 28 subjects from the total sample of 68 patients (41.17%) need hearing aids, either in the form of removeable hearing aids or implants. The mean age at which they begin to use these aids is 18.95 years, with a maximum age of 41 years and a minimum of 1 year (SD = 9.24 years). There are 3 cases of patients with implants, highlighting that two of them required them at an early age due to the appearance of a severe hearing deficit. The rest of the participants use headphones.

With this clinical situation, a linear regression analysis was performed (Table 1, Table 2 and Table 3), enabling the development of a predictive model that would help in the medical analysis of the evolution of the syndrome. It was demonstrated that the “hearing” variable is related to the “sex” variables, as well as the age of DM onset.

As the genetic regions of the WFS1 gene affected in each patient are available, the differences between the different affected regions and the phenotype in terms of the manifested audiological alterations were analyzed. The results verify that the majority of patients who present the homozygous mutation will have a more severe hearing loss phenotype. In fact, only one of the homozygotes did not present a severe phenotype, while two patients with heterozygous alterations presented a severe hearing loss phenotype. This made it possible to establish three groups of genotype–phenotype relationships based on the severity of the phenotypic manifestation.

**Figure 2:**
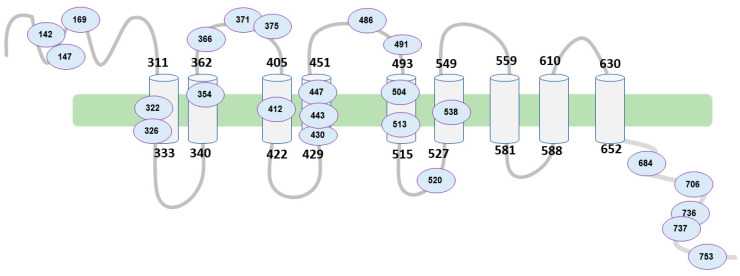
Schematic of WFS1 structure and affected regions. Source: authors’ own elaboration.

Figure 2 represents the structure of the wolframin protein and the affected regions in our study population. The alterations occur both in the regions of the transmembrane segments and in both terminal regions of the protein (hydrophilic and hydrophobic). The patient samples are highly variable in terms of the affected regions of the peptide due to mutations in the WFS1 gene.

Table 4 shows the alterations in the protein with respect to the degree of hearing impairment and the percentage of patients presenting it. The homozygous or heterozygous mutations have been identified, which, in turn, can be duplications, deletions, insertions, loss, or missense. These mutations have been divided into 3 groups in terms of phenotype (Table 4), depending on the severity of the hearing impairment. In the phenotype with severe hearing loss, we found that around 77% of the patients who manifest it present homozygous involvement in exon 8 or exon 4 of the WFS1 gene. On the contrary, those people who present normal or minimally affected hearing are 89% and 91% heterozygous, respectively.

The remaining 11% of patients who showed a normal auditory phenotype present a mutation in homozygosis in exon 8. Specifically, it is located in the C-terminal region of the protein, at amino acid 736, and consists of a change of glycine for serine (p.Gly736Ser). Both amino acids have a polar side chain, which is why they are considered hydrophilic, and the change in the protein does not imply a charge change in said region of the protein.

On the other hand, 9% of the remaining patients with a medium degree of hearing involvement also present the homozygous mutation in exon 8 (c.1113G > A). In this case, this nucleotide change generates a nonsense change in the protein, originating a STOP codon in the sequence. The affected region is located in an extracellular loop between transmembrane domains 2 and 3 of the protein. This mutation was found in a patient with moderate hearing impairment.

Almost 54% of patients with severe hearing loss and with homozygous involvement (77%) share the mutation that occurs in exon 4 of the WFS1 gene, specifically in the c.409_424dup16 coding region. It consists of a 16-nucleotide duplication that generates a frameshift mutation in the N-terminal region of the protein. This alteration supposes the appearance of a STOP-type codon, causing the early termination of the translation of the protein (p.Val142fs*110).

### 3.2. Population 2

In the subgroup of 36 patients, a complete audiological evaluation was carried out to define the type of hearing loss; a longitudinal evaluation of these values was then carried out. A battery of standardized tests with instruments available on the market were used, since there is currently no consensus regarding the study protocol and analysis in an audiological evaluation. Of the 36 patients who attended the multidisciplinary check-ups, 13 (36.11%) presented the 4 main components of the syndrome (diabetes mellitus, diabetes insipidus, optic atrophy, and auditory nerve involvement). A total of 35 patients (97.23%) presented DM and optic nerve atrophy. In 23 patients (63.89%), the first onset symptom was DM, where OA was the first symptom in 25% of the group (9 patients). Only one patient presented sensorineural deafness and an absence of diabetes mellitus from birth.

Specifically, DM occurred in 97.22% (*n* = 35) at a mean age of 6.27 years (SD = 3.67) and ID occurred in 44.44% (*n* = 16) at a mean age of onset of 12 years (SD = 4.29 years). Hearing loss manifested itself in 72.22% (*n* = 26) of the patients, at an onset age of 15.45 years (SD = 8.51 years). In 10 of these patients, the alteration of the auditory nerve was detected before perceiving the hearing loss, presenting alterations only at high frequencies. OA was detected in 100% of the studied population (36 patients), with a mean age of onset of 8.9 years (SD = 4.71 years).

In our measurement, 69.43% of affected patients presented some type of auditory sensory alteration, and that manifestation did not differ substantially between both ears. Patients presenting with a severe or deep hearing loss in both ears represented about 15% of the study population. A higher percentage of patients with a mild hearing alteration (41.6%) was found. In general, there are no significant differences in the auditory alterations present between men and women. No significant relationship was found between the different variables analyzed.

In 2019, high-frequency audiometry was performed on all the patients who attended the assessments. In the same year, the high-frequency test was performed, in which 14 (87.5%) of the 16 patients had high frequency affected. This test was utilized for early detection of WS in 1 patient, whose only history of interest was being the brother of an affected person. This information, together with the fundus examination carried out, caused the researchers to suspect WS, which was later confirmed genetically.

The impedance and tympanometry performed on all the patients, where possible, was determined as normal (100%). It is necessary to make a clarification: in children under 6 years of age, it is not feasible to perform audiometry; therefore, an equivalent test is carried out—auditory evoked potentials. In all the cases in which a patient presents with hearing loss, altered evoked potentials were observed [11].

For patients who indicated impaired swallowing (*n* = 5), a rhinofibrolaryngoscopy (RFL) was performed. Most of these returned normal results, except in one patient, who, in 2021, detected slight paresis of the vocal cords with good glottic closure. We note that he has a tracheotomy, but in the RFL, he presented vocal cords with good glottic space.

In our 36 patients, sensorineural hearing loss (SNH) was assessed using tonal audiometry. It must be clearly differentiated that hearing loss is not the same as audiometry alteration (Table 5). In the determination, the Karzon criterion was followed [17], which is based on the determination of the two following circumstances. The first refers to whether the average value obtained on the conversational scale (500, 1000, and 2000 Hertz) exceeds the value of 20; then, the existence of HN can be suggested. The second refers to the full measurement scale (250, 500, 1000, 2000, 4000, and 8000 Hertz); if, in this measurement, there are more than 2 values that exceed 20 decibels, the existence of HN can be confirmed. When the two circumstances are met, the degree of HN can also be determined.

It was observed (Table 6) that more than 65% of the affected patients presented with some type of auditory sensory alteration and that this manifestation does not differ substantially between both ears. Patients with severe or profound impairment in both ears represented around 15% of the study population. The highest percentage of patients was found to have a mild alteration (around 40%). In general, there are no significant differences in the hearing disorders presented by men and women.

In 2019, high-frequency audiometry was performed on all the patients who attended the assessments. In the same year, the high-frequency test was performed, where 14 (87.5%) of the 16 patients had high frequency affected. This test served us for early detection of WS in 1 patient, whose only history of interest was being the brother of an affected person. The eye fundus examination aroused the suspicion of WS, which was later confirmed genetically.

**Figure 3:**
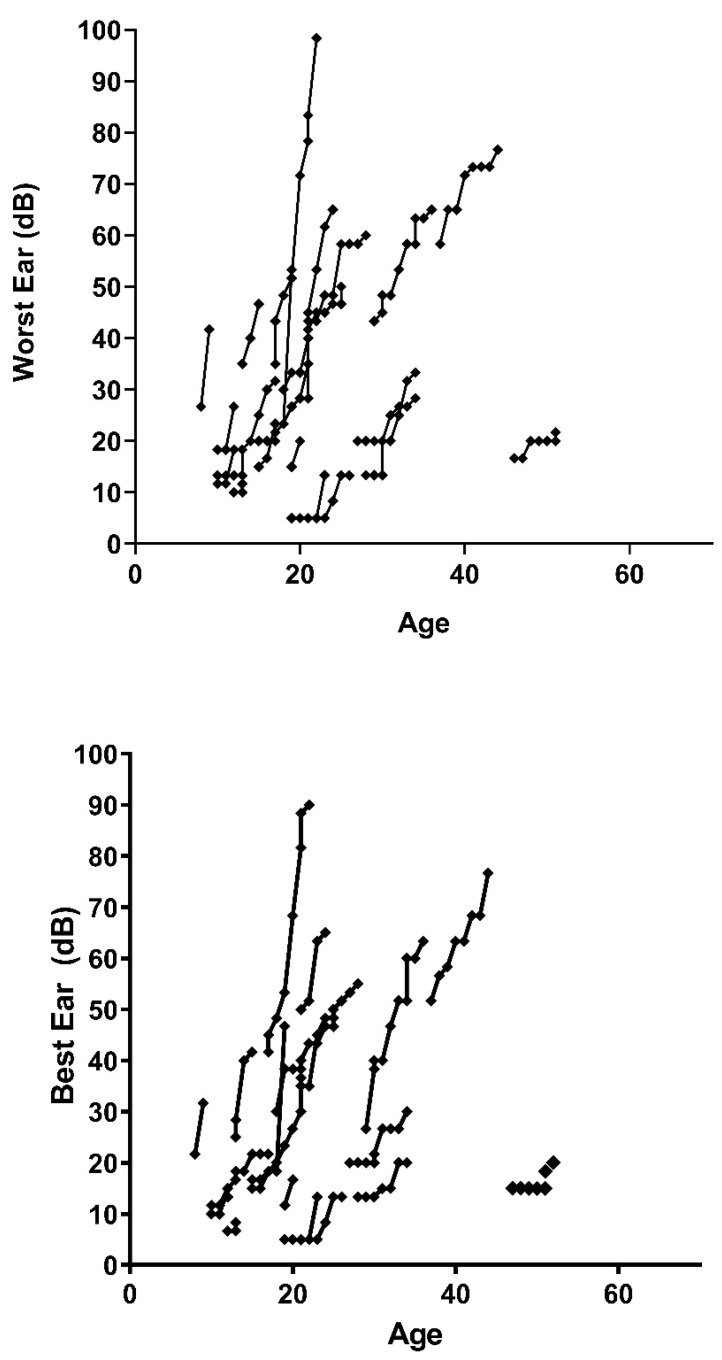
Dispersion of hearing loss data in patients tested as a function of ear tested and age of the individual. Source: authors’ own elaboration.

Figure 3 represents the progression of both ears (worst and best) of the decibels perceived by each patient with respect to their age, the first point being the patient’s age at the first follow-up session. The slope differs between the patients, presenting as practically vertical in some cases. In Figure 2, we can see how some patients present a very vertical slope, close to 90° with respect to the horizontal, while others show much less hearing loss by failing to reach a slope of 45°.

### 3.3. Hearing, CNS, and Mortality

In population 1, there has been a total of 24 deaths (Table 7), 17 of which were due to involvement of the central nervous system at a mean age of 37.5 years (SD 10.04). Four deaths were due to nephro-urological alteration at a mean age of 36 years (SD 5.52) and 3 more were for other reasons. Of the 17 deceased due to involvement of the central nervous system, 16 of them presented a vertiginous picture compatible with audiological involvement. In addition, the peripheral-type vertiginous picture in those who presented it at a mean age of 35.28 years (SD 8.63) manifested it as disabling to carry out their daily life.

## 4. Discussion

For the analyzed population, the altered gene is WFS1, which corresponds to that described in the predominant population in Europe, the United States of America, and Japan [5]. This WFS1 gene encodes a transmembrane protein located in the membranes of the endoplasmic reticulum (ER) and in ER-associated mitochondria [18]. WFS1 is highly expressed in brain tissues, in the beta cells of the pancreas, in the heart, in the lung cells, and in the placenta [5,19]. Mutations in WFS1 cause an accumulation of misfolded proteins in the ER and, therefore, stress on the ER itself [5]. This stress in the ER is the cause of the clinical phenotypes: the DIDMOAD phenotype and sensorineural hearing impairment [20]. This stress on the ER, in turn, has allowed Li et al. to relate WS to Alzheimer’s disease [18], an aspect that still needs to be studied in much greater detail. The sample is consistent with the phenotypes mentioned, since there was a patient who did not present diabetes mellitus. In this sense, this patient is a case of autosomal dominant inheritance whose phenotype is sensorineural hearing impairment, but not DIDMOAD. Bonnycastle et al. determined a non-synonymous variant (p.Trp314Arg) in the Wolfram syndrome 1 (WFS1) gene that completely segregates from the diabetic phenotype [20]. In our case, the allele is the p.Ala684Val present in the WFS1 gene, which, phenotypically, also separates from the DIDMOAD phenotype. This extreme complexity, which has already been pointed out previously [21], makes it difficult to clearly elucidate its specific pathogenesis. In fact, we agree with Ren et al. when they stated that further functional studies of genes and proteins are lacking [21].

Regarding the debut in population 1, we observed, as in other published series, that the most common symptoms is DM, followed by AO [22,23]. However, in this study, it was observed that two patients presented with deafness from birth, which is attributable to the fact that one case is autosomal dominant and the other presents a more severe affectation, since it was associated with a cytomegalovirus infection during pregnancy. It was highlighted that one patient presented with isolated DI and three patients presented with DM associated with DI without other symptoms, which, based on previous literature, is highly improbable. In these four cases, type 1 DM also manifested itself later and none presented with this symptom after the age of 18, which is in agreement with previous studies that indicate that type 1 DM is a child–adolescent age disease [1,15]. When analyzing the clinical data of these patients, there was no detection of a significant differentiating element, except for the relationship between the age of DM and gender. This is because, as indicated by Delvecchio et al. [23], the clinical history can be different, even between patients who are in the same pedigree.

Wolframin deficiency affects neuronal survival, which leads to hearing problems. In the case of WS, the most affected areas are the sensory pathways, the brainstem, the cerebellum, and the hypothalamus [24,25]. In the specific case of auditory pathways, the effects are multiple. In fact, among other areas, effects have been verified in both the organ of Corti and the cochlear nerve, and even in balance [24,25]. As confirmed by the results, these phenotypic manifestations are not constant. However, it cannot be ruled out that the homozygous mutation, over time, may lead to greater hearing loss.

On the other hand, various authors have identified mutations in the loop regions of the wolframin protein that cause variable phenotypic manifestations with different degrees of involvement [26]. Vanita Berry et al. [26] reported a mutation in a loop protein (c.A1385G; p.E462G) causing a congenital nuclear cataract as a relevant manifestation in one family. In turn, Munshani Saira et al. [27] reported different mutations (A559T, E394V, and R558C) associated with psychiatric symptoms of differing impact, which seem to highlight the relevance of the protein loops located at the interface between the cytoplasm and the membrane. In our case, the most severe cases occurred in exon 4 of the WFS1 gene. This partially agrees with Zhao et al. [28], since these authors also detected that the mutation in exon 4 shows a profound or severe affectation phenotype. In contrast, it has been detected that mutation in exon 4 may not alter hearing loss [29]. However, most mutations occur in exon 8 [28,30], which, again, shows the complexity of the WS.

. In this syndrome, it must be taken into consideration that the cerebellum is also significantly affected, which can cause an increase in the impact of vertigo of peripheral origin. A recent study suggests that there are two distinct histopathologic abnormalities associated with WS. The first is neuronal loss and gliosis in limited cortical and subcortical gray matter, and the second is patchy demyelination and axonal degeneration in various white matter tracts [25]. This pattern ranges from myelin and axonal loss to generalized myelin and axonal and neuronal loss [31].

It has been suggested that when the cases are ordered according to the age of the patient and the severity of the disease, the evolution of the neuropathological alterations follows a specific pattern [30], which is consistent with what was found in our sample. It was not possible to perform histopathological studies on all the patients after death, as it is an invasive post-mortem test. In this sense, it should be noted that it is difficult to obtain permission from the family so that, at the time of death, the anatomopathological study can be carried out, given the great emotional impact it entails.

Regarding the qualitative variable “hearing”, which aims to show some hearing dysfunction, the evolution of population 1 is statistically related to the sex and age at which the patients presented with type 1 DM. In contrast, in population 2, it was not possible to establish statistical correlations between the different variables. In our results, there was not a verification of a relationship between the manifestations of neuronal disorders and hearing loss, but the existing data could suggest this relationship. After all, there are great difficulties in establishing a clear and determined pattern of this condition. All this complexity and variability that has been mentioned shows us a clinical and genetic complexity with heuristic difficulties.

Given the evolution of the disease indicated in the results, it is especially important that an exhaustive follow-up be carried out on those affected for the early detection of all the entities that make up the syndrome. In this sense, it is important to note that patients with homozygous genetic alterations will have a more severe hearing loss phenotype. Hence, it is important to determine the genetic characteristics in patients diagnosed or suspected of presenting WS.

The results for population 1 allowed an indication that hearing loss is more frequent after 18 years of age. On the other hand, thanks to the analysis of population 2, and taking into account the vertical evolution of hearing loss, the results allow the suggestion of periodic audiometry tests, at least once a year, to medical professionals. The rationale is to improve counseling and appropriate treatment [17], and upon detection of hearing loss, it will be easier for patients to adapt to hearing aids. This aspect is important for patients facing difficulties with socialization, school integration and, therefore, an adequate career path. However, as the analyzed information confirms, there is a delay in the diagnosis of WS, which implies a delay in the detection of the hearing alteration. This fact could increase these problems of social integration.

In our study, all the patients who had hearing impairment had neurosensory affectation and also presented bilateral and symmetrical hearing loss, which is similar to the results indicated in other studies [16,17]. For this reason, it is important to consider the performance of high-frequency audiometry, since this syndrome has been correlated with hearing loss at higher frequency levels [32]. The reported results are consistent with this study and suggest that younger patients tend to see their hearing capacity more likely to be affected at this frequency level, while older patients less so. This aspect should be studied in greater detail to contrast it.

The main limitations of this study are that the sample is mainly limited to Spain and Portugal. Although the study is deep at the temporal and analytical level, the data cannot be generalized to all people affected by WS. Likewise, our sample is made up of people affected by WS1; therefore, we have not been able to analyze people affected by type 2 or by WSL. Despite these limitations, the authors of this study consider this research to provide valuable information for future studies of this condition.

## 5. Conclusions

This is the first study to analyze the hearing impairment of patients affected by WS, mainly in the Iberian Peninsula area. This research also delves into the temporal evolution of hearing impairment in the population under study. In this sense, the study increases the audiological knowledge of WS and complements previous works. In addition, in this investigation, it has been verified that there is a statistical relationship between the qualitative variable hearing with sex and the age of onset of DM. This aspect needs further investigation, but it could be of great interest for the follow-up of diagnosed patients.

On the other hand, hearing impairment and its progression vary greatly from one patient to another with WS; therefore, a fixed pattern cannot be established. However, it is possible to affirm that those with homozygous genetic alterations will have a phenotype with greater severity of neurosensory involvement. This makes it possible to determine a clear follow-up and control factor in patients. In addition, it also leads us to rely on routine genetic analysis when there is suspicion of WS.

An early suspicion of WS would promote adequate monitoring of all the entities that make up the syndrome, especially hearing loss, which usually begins to be monitored when it is already very evident. A standardized protocol would make it possible to apply hearing aids or cochlear implants early, which would promote greater social integration in all areas (education, work, etc.).

Hearing loss associated with a loss of vision is one of the manifestations that patients indicate as the most limiting in their daily lives, to which must be added that the symptoms associated with the affectation of the auditory nerve produce a dizzying condition that incapacitates the patient. Therefore, the development of therapies that slow down or improve this affectation of the auditory nerve is important.

Early detection of hearing problems, as well as WS itself, is very important since, in our case, it has allowed the close monitoring and treatment of the affected person from very early stages. On the other hand, and given the results obtained, the authors of the paper believe that it would be advisable in those children born with sensorineural deafness to undergo a genetic analysis of deafness in which WS is included for early detection and treatment.

## Figures and Tables

**Table 1:** Summary of model ^b^.

Model	R	R^2^	R^2^ Ajusted	Standard Error of the Estimation	Statistics of Change					
					Change in R^2^	Change in F	df1	df2	Sig. Change in F	Durbin-Watson
1	0.995 ^a^	0.991	0.990	0.647	0.991	3391.563	2	65	0.000	1.949

Source: authors’ own elaboration. Notes: ^a^ Predictors: (Constant), age of DM, sex. ^b^ Dependent variable: hearing.

**Table 2:** ANOVA ^a^.

Model		Sum of Squares	Df	Root Mean Square	F	Sig.
1	Regression	2835.811	2	1417.905	3391.563	0.000 ^b^
	Waste	27.174	65	0.418		
	Total	2862.985	67			

Source: authors’ own elaboration. Notes: ^a^ Dependent variable: hearing. ^b^ Predictors: (Constant), age of DM, sex.

**Table 3:** Coefficients ^a^.

Model		Non-Standardized Coefficients		Standardized Coefficients	T	Sig.	Collinearity Statistics	
		B	Dev. Error	Beta			Tolerance	VIF
1	(Constant)	−0.847	0.117		−7.235	0.000		
	Sex	1.215	0.039	0.920	31.124	0.000	0.167	5.984
	Age of DM	0.066	0.024	0.082	2.767	0.007	0.167	5.984

Source: authors’ own elaboration. Notes: ^a^ Dependent variable: hearing.

**Table 4:** Regions of affected protein in the sample, along with percentage and severity ranking. Note: fs indicate frame shift, and * indicates that the protein ends prematurely. Source: authors’ own elaboration.

Affected Protein Region		Percentage %	Hearing Loss Classification
p.Glu737Lys	p.Tyr706X	22.2	Normal
p.Val142fsX110	p.Glu753X	11.1	
p.Gly736Ser	p.Gly736Ser	11.1	
p. Val142Glyfs*118)	p.Ser 443 Arg	11.1	
p.Arg375His	p.Val412SerfsX	22.2	
p.Gln366 X	p.Phe538 Leu	11.1	
P. Val412Ser fs*29	p. Val491_P792insLITV	11.1	
p.Phe354del	p.Gly736Ser	7.1	Mild
p.Tryp371X	p.Trp371X	7.1	
p.Phe354del	p. Val491_P792insLITV	21.4	
p.Glu169GlyfsX119	p.Pro504Leu	14.3	
p.Val142Glyfs*110	p.G736S	7.1	
p.(Ala326Val)	p.(Gln486Leufs*57)	7.1	
p.Ser430X		7.1	
p.Val142fsX110	p.Tyr 706X	7.1	
p.Val412Ser fs*29	p.Val412Ser fs*29	7.7	Profound
p.v142gfs*110	p.R147P	7.7	
p.Gln520X	p.Gln520X	7.7	
p.Tyr706X	p.Tyr 706X	7.7	
p.His322fsX	p.Val509_Tyr513del5	7.7	
p.Ala684 Val		7.7	
p.Val142fsX110	p.Val142fsX110	53.8	

**Table 5:** Scheme of the conversational scale.

Mean on the Conversational Scale (Decibels)	Classification
21 to 40	Mild
41 to 70	Moderate
71 to 90	Severe
91 to 120	Deep
>to 120	Cofosis

Source: authors’ own elaboration.

**Table 6:** Classification, percentage, and frequency of hearing disorders divided according to ear.

	Right Ear Hearing Loss		Left Ear Hearing Loss	
Classification	Percentage	Frequency	Percentage	Frequency
Normal	30.55%	11	30.55%	11
Mild	41.66%	15	38.88%	14
Moderate	11.11%	4	16.66%	6
Severe	11.11%	4	5.55%	2
Deep	5.55%	2	8.33%	3

Source: authors’ own elaboration.

**Table 7:** Summary of causes of death of deceased patients.

Central Nervous System					
Bronchoaspiration (*n* = 4)		Central respiratory failure (*n* = 10)		Others (*n* = 3)	
Females (*n* = 2) - 42 years - 30 years	Males (*n* = 2) - 34 years - 43 years	Females (*n* = 5) - 37 years - 39 years - 25 years - 48 years - 41 years	Males (*n* = 5) - 39 years - 29 years - 61 years - 47 years - 40 years	Females (*n* = 0)	Males (*n* = 3) - 28 years: Hypoglycemic encephalopathy. - 31 years: Cerebral Hemorrhage. - 16 years: Laryngeal spasm of central cause.
Urological Cause				Other causes	
Renal Insuffiency (*n* = 3)		Urinary sepsis (*n* = 1)			
Females (*n* = 0)	Males (*n* = 3) - 42 years. - 38 years. - 37 years.	Females (*n* = 1) - 27 years.		Females (*n* = 3) - 60 years (Uterine cancer) - 20 years (Pulmonary hemorrhage) - 31 years (Leukemia)	

Source: authors’ own elaboration.

## Data Availability

The data reported here are available at request by scientific community members.
